# The Synergistic Effect of Immune Checkpoint Blockade and Radiotherapy in Recurrent/Metastatic Sinonasal Cancer

**DOI:** 10.7759/cureus.3519

**Published:** 2018-10-29

**Authors:** Gozde Yazici, Ibrahim Gullu, Mustafa Cengiz, Aysenur Elmali, Melek Tugce Yilmaz, Secan Aksoy, Sezin Yuce Sari, Gokhan Ozyigit

**Affiliations:** 1 Radiation Oncology, Hacettepe University Medical School, Ankara, TUR; 2 Oncology, Hacettepe University Medical School, Ankara, TUR; 3 Radiation Oncology, Hacettepe University Medical School, Ankara, USA; 4 Oncology, Hacettepe University Cancer Institüte, Ankara, TUR

**Keywords:** maxillary sinus cancer, stereotactic radiotherapy, immune checkpoint blockage

## Abstract

Treatment options for recurrent/metastatic sinonasal cancer (RMSNC) patients are limited. We present two cases with RMSNC treated with a combination of immune checkpoint blockade and hypo-fractionated stereotactic radiotherapy (HSRT).

Case 1 presented with RMSNC three months after the primary treatment. The patient progressed under first-line chemotherapy and pembrolizumab was offered. The disease progressed after the sixth cycle. We performed reirradiation with HSRT to the primary site. Case 2 presented with local recurrence eight years after the primary treatment for maxillary sinus cancer. He refused surgery and chemotherapy and was offered nivolumab treatment. After two doses, we performed reirradiation with HSRT.

Case 1 showed regression at both the local and the metastatic sites after radiotherapy. The second patient’s symptoms resolved completely three months after radiotherapy.

The HSRT and immune checkpoint blockade combination is a promising treatment option for patients with RMSNC.

## Introduction

Sinonasal cancers represent 5% of all head and neck cancers. The most common subset is the maxillary sinus and the most common histology is squamous cell carcinoma (SCC) [1]. The recommended primary treatment is surgery, and early-stage disease (stages I and II) is treated with a single-modality treatment. In the locally advanced stage, despite aggressive treatments, the predominant pattern of failure and the main cause of death is local recurrence [1-2]. Treatment options for recurrent/metastatic sinonasal cancer (RMHNC) patients are limited and the prognosis is poor. First-line treatment with platin-based chemotherapy and cetuximab yields an overall survival of 10 months [3]. However, there is no standard second-line treatment option for patients who progress after this treatment.

Immune checkpoint blockade (ICB) has emerged as a promising treatment option. Numerous anti-PD-1 and anti-PD-L1 antibodies are under investigation, and two of them (nivolumab and pembrolizumab) were approved by the Food and Drug Administration (FDA) in 2016 for patients with RMHNC [4-5]. Pembrolizumab (MK-3475) and nivolumab are monoclonal antibodies directed against the programmed cell death protein 1 (PD-1), which is expressed by activated T cells. PD-1 and its ligands (PD-L1 and PD-L2) are mainly involved in the modulation of T-cell activity in peripheral tissues. As a result of the interaction of PD-1 with its ligands PD-L1 and PD-L2, T-cell activity is repressed. Tumors upregulate PD-L1 to mediate immune tolerance. Blocking the PD-1 pathway enhances T cell-mediated killing of the tumor cells.

While ICB has led to impressive results against some chemotherapy- and radiotherapy-resistant tumors, an objective response is observed in approximately 20%-40 % of patients [6]. In addition, many progress after an initial response due to the development of secondary resistance. The combination of ICB and stereotactic radiotherapy with ablative doses (8-10 Gy) is a field of ongoing investigation, and studies show that radiotherapy may act as an immunologic booster by potentiating and modulating tumor immunity [7-8].

In this report, we present two cases of recurrent and/or metastatic maxillary sinus SCC treated with a combination of pembrolizumab and hypo-fractionated stereotactic radiotherapy (HSRT) with excellent outcomes.

## Case presentation

Case 1

A 23-year-old male with no previous history of disease was admitted to the hospital with complaints of swelling, pain, and loss of sensation on the right side of the face. Physical examination and magnetic resonance imaging (MRI) revealed a mass extending from the inferior orbital rim to the gingivobuccal sulcus. A biopsy was performed, and the pathological examination was reported as poorly differentiated carcinoma with squamous differentiation (compatible with NUT midline carcinoma). Positron emission computed tomography (PET-CT) showed fluorodeoxyglucose (FDG) uptake in the bilateral level II and III lymph nodes and right anterior maxillary sinus. He underwent total maxillectomy and right radical neck dissection. Pathology revealed poorly differentiated carcinoma with squamous differentiation (compatible with NUT midline carcinoma). The tumor was 4 cm in the largest dimension, with perineural invasion. The resection margins were tumor positive, and the dissected 46 lymph nodes were negative for metastases.

**Figure 1:**
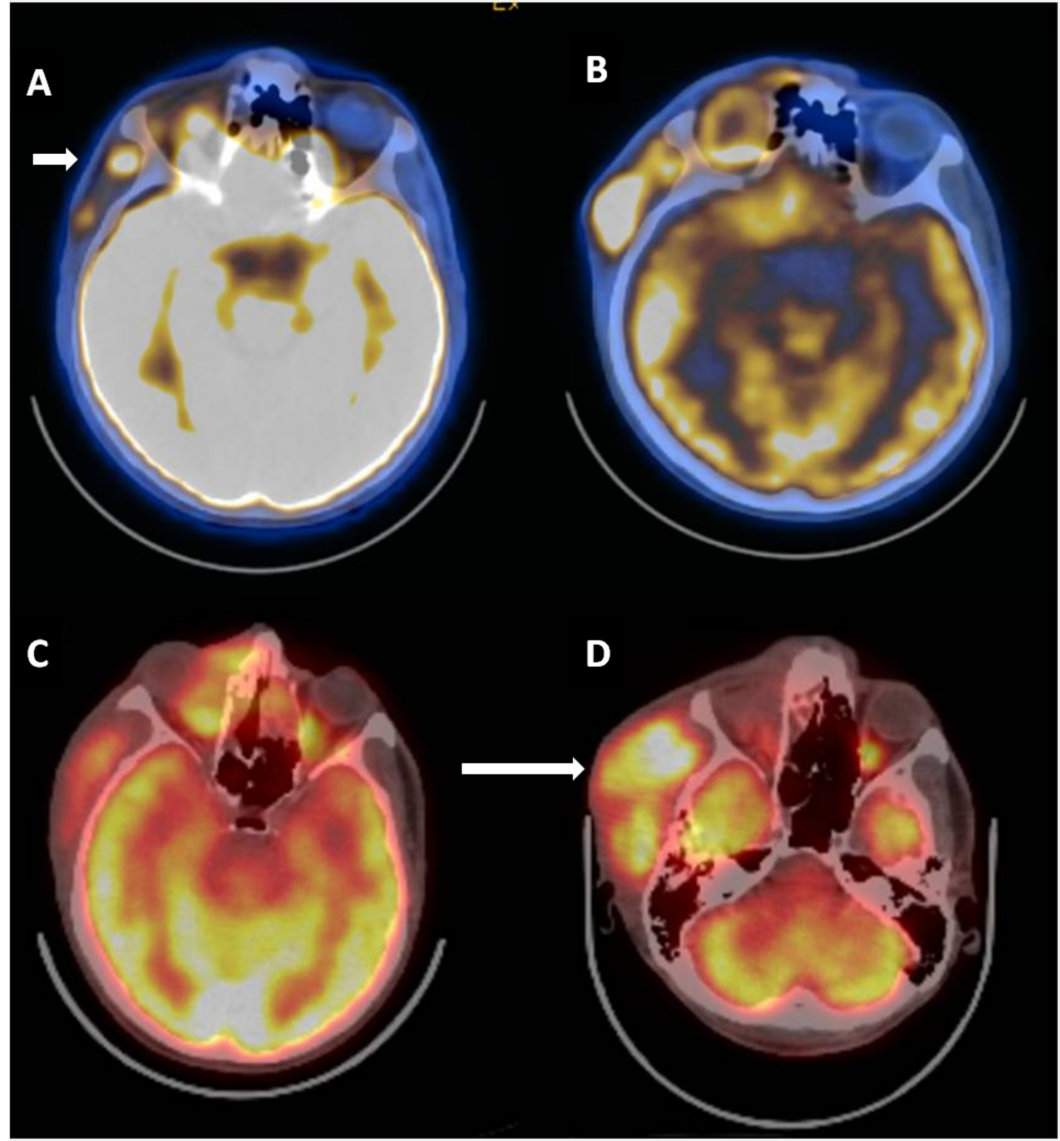
Positron emission computed tomography (PET-CT) performed before, during, and after chemotherapy for recurrence showing increased fluorodeoxyglucose (DG) uptake 1A. PET-CT performed three months after primary radiotherapy showing an FDG avid recurrent lesion (short arrow) in the right pterion and right orbit and in the lateral side of the zygomatic bone. 1B. PET-CT performed after two cycles of docetaxel/ cisplatin and six cycles of gemcitabine in combination with oxaliplatin showing the progressive lesion. 1C. PET-CT performed after four cycles of pembrolizumab showing a partial response in the primary site. 1D. PET-CT performed after eight cycles of pembrolizumab showing progressive disease (long arrow).

Postoperatively, he underwent adjuvant concomitant chemoradiotherapy. The prescribed doses were 66 Gy to the primary site and 57 Gy to the right neck levels in 33 fractions, with weekly cisplatin. The treatment was delivered using intensity modulated radiotherapy (IMRT) and it was well-tolerated with moderate acute side effects like grade 1 mucositis and dermatitis. A follow-up MRI scan three months after radiotherapy revealed a recurrent lesion in the right pterion and right orbit, 16 mm in the largest dimension. The PET-CT showed increased metabolic activity in the lateral side of the zygomatic bone, in the right spina scapula and right acetabulum, which were evaluated as bone metastases (Figure 1A). He received two cycles of docetaxel/cisplatin and six cycles of gemcitabine in combination with oxaliplatin (GEMOX); however, the PET-CT showed progressive disease with increased FDG uptake in the right medial orbit, right zygomatic bone, right acetabulum, right pubic ramus, right lung, and cervical lymph nodes as revealed in Figure 1B and in data not shown.

**Figure 2:**
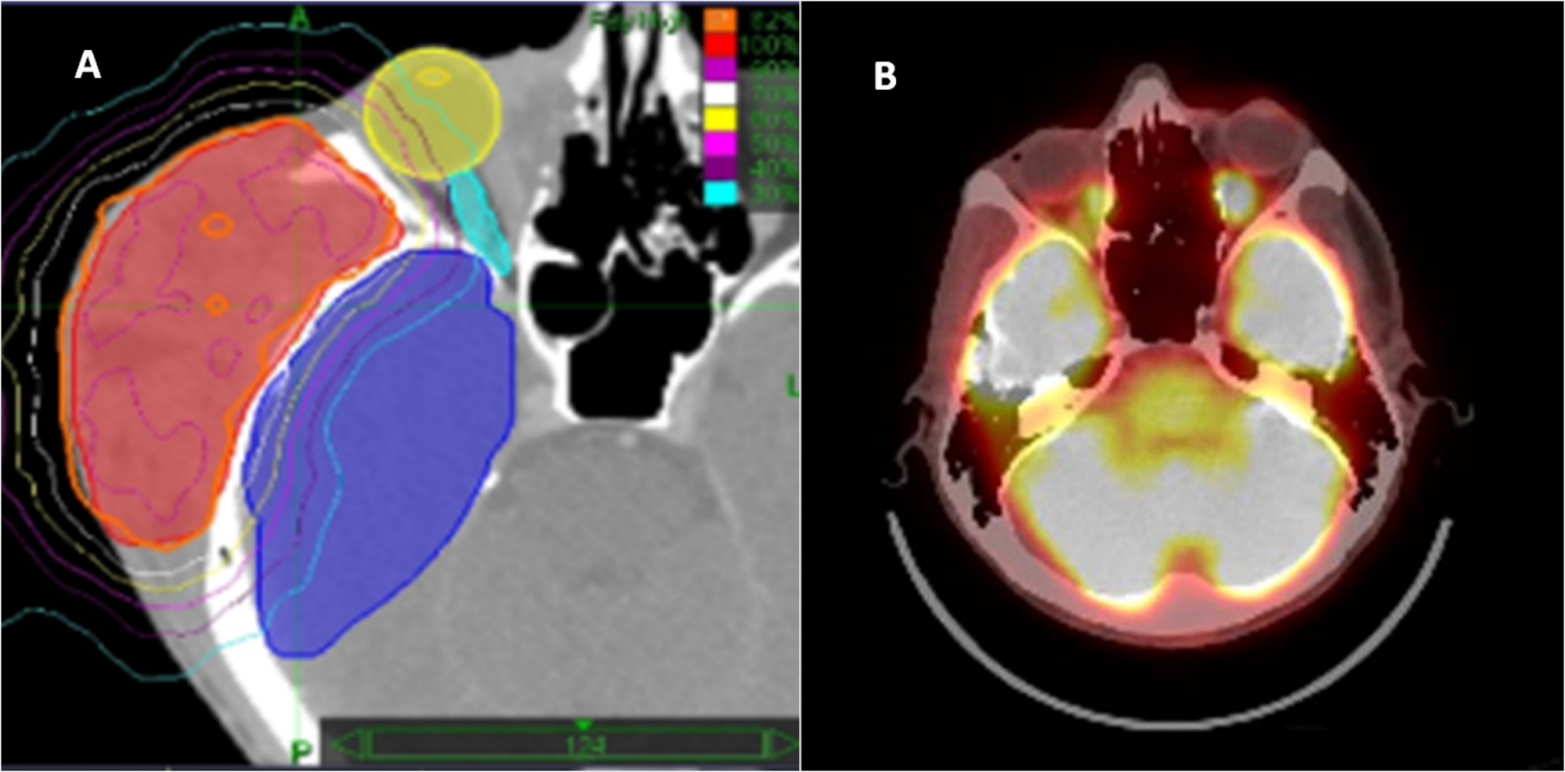
Radiotherapy treatment plan and the response to radiotherapy 2A. The hypofractionated stereotactic radiotherapy (HSRT) plan showing the lesion covered by the 95% isodose line. The dark blue area showing the right temporal lobe, the yellow area showing the right eye, and the light blue area is the right optic nerve. 2B. Positron emission computed tomography performed eight months after HSRT showing a complete response at the irradiated site.

As a second-line treatment option, he was offered pembrolizumab. After four cycles of pembrolizumab, he had both a clinical and a radiological partial response (Figure 1C). The FDG uptake in the right zygomatic region and right maxillary region decreased. However, the response was not durable and the soft tissue mass progressed clinically after the sixth cycle. The PET-CT performed after the eight cycles showed progression in the soft tissue lesions in the right orbital and zygoma and the right scapula (Figure 1D). We performed HSRT with a total dose of 24 Gy, in three fractions to the orbital mass (Figure 2A). The fractions were delivered every other day. He continued pembrolizumab treatment after radiotherapy. Clinically, the tumor responded to radiation. Repeat imaging performed three months following the completion of radiation therapy showed a complete response in the orbital mass and in the metastatic lesion in the left sixth rib (Figure 2B). There was a significant reduction in the FDG uptake of the right acetabulum and right ischium. However, the right scapular lesion did not respond to treatment. We performed the same radiotherapy dose (24 Gy in three fractions) to this lesion and the follow-up imaging studies showed a complete response in all FDG avid areas.

Case 2

A 29-year-old male patient presented with complaints of exophthalmos and swelling in his right eye, which started one month ago and increased gradually. After a detailed history and physical examination, a paranasal sinus CT and nasopharynx MRI was obtained. The images revealed a solid mass, approximately 4x3.4x4.1 cm in size. The right maxillary sinus was obliterated with the lesion, which also invaded the sphenoid bone. It extended into the middle crust in the nasal cavity and to the orbital cavity, eroding the bony structures of the apex and the medial wall of the orbit but not infiltrating the orbital muscle tissues. There was also an inferior temporal fossa extension. A biopsy was performed and the pathology was reported as a "basaloid type SCC." He refused surgery and received three cycles of docetaxel, cisplatin, and 5-fluorouracil (DCF) induction chemotherapy. The imaging studies showed minimal response to chemotherapy.

**Figure 3:**
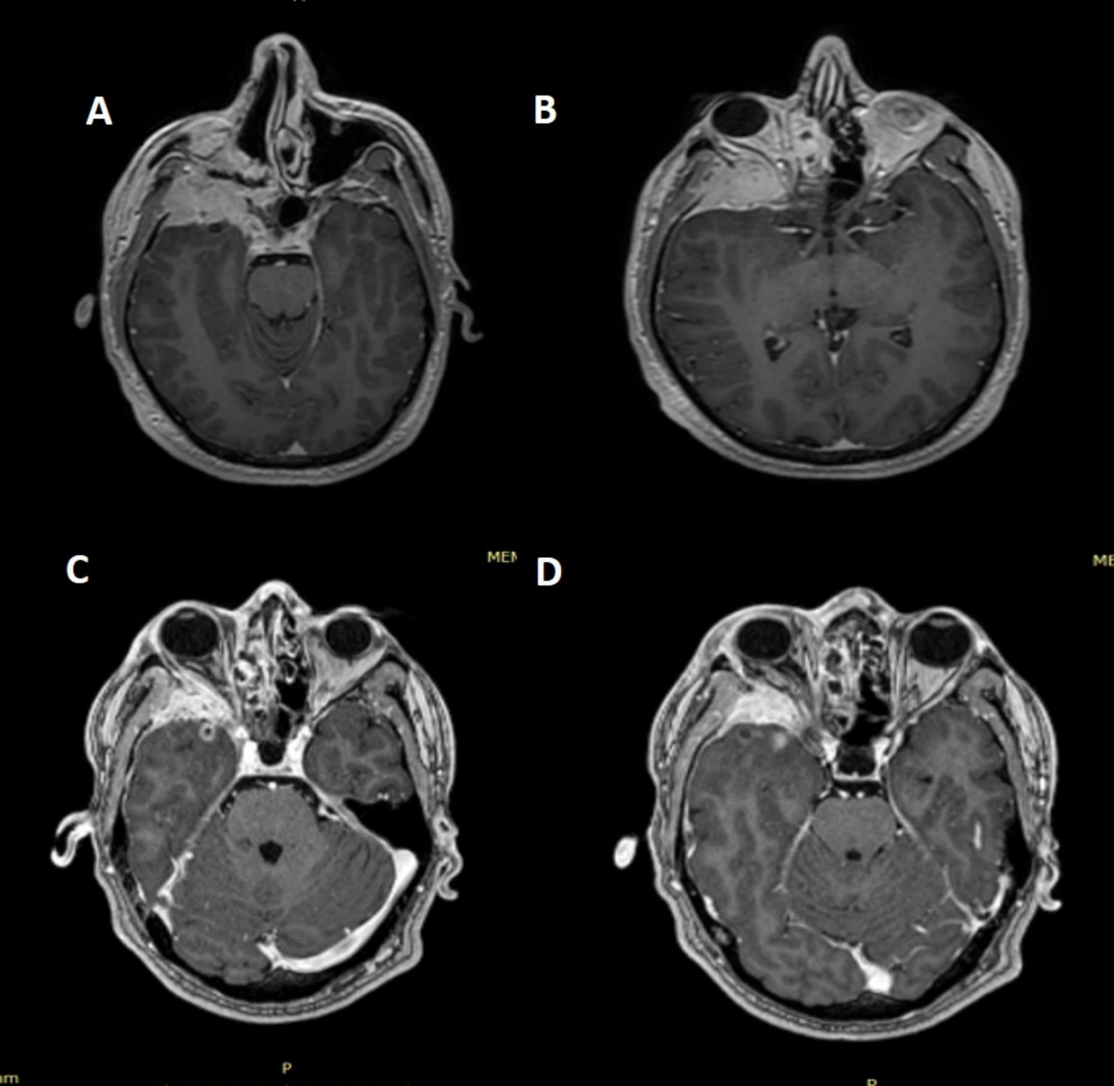
Magnetic resonance images (MRIs) before and after hypofractionated stereotactic radiotherapy 3 A-B: MRI at recurrence 3 C-D: MRI four months after radiotherapy

He received HSRT to a total dose of 36 Gy with 7.2 Gy/fraction to the primary site. Follow-up images revealed minimal regression. He was followed without any medication with MRI performed every three months, and the disease was stable. Eight years after radiotherapy, the MRI showed progression in the intracranial and infraorbital compartments and the patient had a complaint of progressive exophthalmos. The MRI revealed a lesion extending into the orbit and the optic nerve swirled all the way, the temporal lobe compartment infiltrated the large wing of the sphenoid sinus, the cavernous sinus, and the Meckel cavity (Figures 3A-3B). There was also dural infiltration in the temporal region. He refused surgery or chemotherapy and was offered nivolumab. After two cycles, we performed HSRT to the locally recurrent lesion with a fraction dose of 8 Gy to a total dose of 24 Gy, every other day. He is still receiving nivolumab and the follow-up images performed four months after radiotherapy revealed a significant response, and his complaint of exophthalmos resolved completely (Figures 3C-3D).

## Discussion

Treatment options in RMHNC are limited. Standard chemotherapy regimens are used to achieve palliation, and the overall survival rates are around 10 months. In this case report, we presented two maxillary sinus cancer patients, one with both local recurrence and distant metastases and the other with only local recurrence. Case 1 was heavily treated with chemotherapy prior to ICB. Case 2 received only induction chemotherapy before primary radiotherapy and refused additional chemotherapy after local progression. The patients received ICB and the HSRT was performed between the cycles. We achieved durable local responses in both cases, without any significant side effect.

CheckMate 141 (ClinicalTrials.gov, NCT02105636) evaluated the efficacy, safety, and patient-reported quality of life of nivolumab monotherapy vs standard single agent of investigator’s choice (IC) in patients with RMHNC who experienced tumor progression or recurrence within six months of platinum-based therapy [5,9-10]. First publications reported a survival advantage with nivolumab [5]. With long-term follow-up (24.2 months), nivolumab nearly tripled the estimated 24-month overall survival (OS) rate (16.9%) vs IC (6.0%), and demonstrated OS benefit across patients with tumor PD-L1 expression ≥1% and <1%, regardless of tumor human papillomavirus (HPV) status [10]. Both patients with HPV-positive and HPV-negative tumors benefited from nivolumab therapy. However, none of the patients in this study had a nasal cavity or paranasal sinus primary. Our second case received nivolumab before and after HSRT and achieved a response that was not achieved in the primary treatment.

KEYNOTE-012 study (ClinicalTrials.gov, NCT01848834) is a phase 1b study of pembrolizumab treatment in RMHNC [11]. In this study, 192 patients were enrolled and confirmed that the objective response rate (ORR) was 17.7%. Thirty-three (17%) patients achieved stable disease. ORR was seen in 21.9% of HPV positive and in 15.9% of HPV negative patients. The median overall survival was 8.5 months. These were patients who were heavily pretreated and a majority of them had more than two lines of previous therapy. Our first case was also heavily pretreated with chemotherapy prior to pembrolizumab. The response to pembrolizumab was transient, but the HSRT acted as an immunologic boost and there was complete response following radiotherapy.

A randomized phase III trial (Keynote 040) (ClinicalTrials.gov, NCT02252042) compared pembrolizumab monotherapy versus IC for advanced HNSCC [12]. Increase in OS over IC systemic therapy was not reached. However, responses were higher and more durable when compared to IC and the risk of death was reduced by 19% in the pembrolizumab arm. The use of immunotherapy after progression in the control arm could explain the lack of a survival benefit in the pembrolizumab group. In this patient pool, there were only three patients with sinonasal cancer.

There are no validated biomarkers for the prediction of response to ICB in RMHNC patients. However, it is known that higher tumor PD-L1 expression levels and a higher mutational burden are correlated with a higher response rate to PD-1-based immunotherapy. Riobello et al. studied the PDL-1 expression in 53 sinonasal cancer patients [13]. They observed PD-L1 positivity in infiltrating immune cells in 45% of patients, and 34% of patients showed PD-L1 positivity on tumor cells. They concluded that this may support the use of ICB in patients with sinonasal cancer.

It was shown that cell killing by ionizing radiation contains an immune-mediated component [8]. The radiotherapy dose required to control tumor growth in mice that were T cell competent was 50% lower as compared to those that were T cell depleted. Several studies demonstrated the connection between the abscopal effect of radiotherapy and the immune system.

Radiation causes immunogenic cell death in tumors by liberating neoantigens. This stimulates a tumor-specific immune response, with activation of antigen-presenting cells and then CD8+ T cell priming. The CD8+ T cells can recognize both the primary tumor and the metastatic sites [14]. Radiotherapy also causes increased accumulation of primed CD8+ T cells in the tumor microenvironment. However, not all effects of radiotherapy effect the immune system positively. Radiation causes also immune- suppression by increasing the PD-L1 receptors that inhibit cytotoxic effect [7]. The limited number of cases with the abscopal effect of radiotherapy up to date may be a result of the dual effect (stimulant/ inhibitory) of radiotherapy on the immune system [14]. In case of a combination of ICB with radiotherapy, this suppressor effect of radiation can result in the increased efficacy of the ICB [8]. There are a lot of unanswered questions regarding the timing of radiotherapy, timing of ICB and radiotherapy, best site for treatment in metastatic cases, and so on. But Vanpouille-Box et al. published a novel study showing that exonuclease TREX1 abrogated the immunogenicity of irradiated cancer cells by degrading interferon-stimulatory cytosolic dsDNA [15]. TREX1 upregulation by radiation dose per fraction beyond a threshold of 10-12 Gy resulted in poor synergy with immune checkpoint blockers. They also demonstrated that 24 Gy in three fractions scheme seems to be stimulating the immune response more efficiently compared to lower doses as well as one fraction-based scheme such as 20 Gy in one fraction. Based on the findings of this preclinical study, we have given a radiotherapy dose of 24 Gy in three fractions. To the best of our knowledge, these two cases are the first clinical evidence supporting the findings of Vanpouille-Box et al.

## Conclusions

To the best of our knowledge, these cases are the first maxillary sinus cancer patients treated with ICB and radiotherapy with dramatic responses reported in the literature. When used alone, the radiation dose used to treat the recurrent tumors would not be expected to control the lesions. We obtained dramatic responses with the combination of immunotherapy and 24 Gy in three fractions HSRT. This particular combination schedule is worthy of testing in future prospective clinical trials.
